# The Impact of Nutritional Supplementation During Pregnancy on the Incidence of Gestational Diabetes and Glycaemia Control

**DOI:** 10.3389/fnut.2022.867099

**Published:** 2022-04-08

**Authors:** Ibrahim Ibrahim, Mohammed Bashir, Parul Singh, Souhaila Al Khodor, Hala Abdullahi

**Affiliations:** ^1^Sidra Medicine, Weill Cornell Medical College-Qatar, Doha, Qatar; ^2^Endocrine Department, Hamad Medical Corporation, Doha, Qatar; ^3^Research Department, Sidra Medicine, Doha, Qatar; ^4^College of Health and Life Sciences, Hamad Bin Khalifa University, Doha, Qatar

**Keywords:** pregnancy, gestational diabetes, Myo-Inositol, probiotics, vitamin D, fish oils, omega 3

## Abstract

The nutritional state before and throughout pregnancy has a critical impact on the women's health and the baby's development and growth. The release of placental hormones during pregnancy induces/ increases maternal insulin resistance and promotes nutrition utilization by the fetus. Gestational Diabetes Mellitus (GDM) is the most common medical complication in pregnancy and is associated with significant maternal and fetal morbidity. Several studies have examined the effect of physical activity, healthy eating, and various food supplements on the risk of developing gestational diabetes (GDM) and related outcomes. Among those, Myo-Inositol supplementation has shown encouraging results in the prevention of GDM. Maternal vitamin D deficiency has been associated with an elevated risk of GDM, and supplementation can improve glucose haemostasis by lowering fasting blood glucose, HbA1c, and serum insulin concentration. Probiotics modulate the gut microbiota leading to an improved glucose and lipid metabolism, which is proposed to reduce the risk of GDM. We aim to review the strength and limitation of the current evidence for using some nutritional supplements either as single agents or in combinations on the risk of developing GDM and on glycaemic control.

## Introduction

Appropriate nutritional health before and during pregnancy is essential for favorable outcomes and the long-term health of the offspring. One or more nutritional deficits in mothers before and in early pregnancy are not uncommon and increase adverse pregnancy outcomes. Gestational diabetes mellitus (GDM) is a common pregnancy disorder associated with an increased risk of pregnancy complications and long-term metabolic complications for both mothers and offspring (1). Pre-eclamptic toxemia, preterm labor, large for gestational age, neonatal hypoglycaemia, and cesarean delivery are serious pregnancy complications associated with GDM. Besides, GDM increases the future risk of hypertension, type 2 diabetes (T2DM), fatty liver disease, and cardiovascular disease in women and offspring (1).

Dietary patterns before conception and during pregnancy are associated with a reduced risk of GDM. On the one hand, increasing the intake of vegetables, fruits, whole grains, nuts, legumes, and fish can lower the risk of GDM (2). While, high consumption of red meat, processed meat, and eggs can increase GDM risk (3). Dietary screening questionnaire tools can identify these nutritional habits in early pregnancy and implement tailored feedback interventions (4). Dietary counseling is the cornerstone in the treatment of GDM, and all women with GDM should be offered dietary advice by a trained clinical dietician (5).

Maternal obesity is the most critical and modifiable risk factor of GDM. While obesity is a synonym for overnutrition, obese people have higher micronutrient deficiencies than normal-weight individuals (6, 7). Most critical, some nutritional deficiencies might exacerbate insulin resistance in obese women leading to increased risk of GDM or worsening of glycaemic control in women with GDM. Hence, it is vital to understand the impacts caused either by deficiency or excess of some micronutrients on the risk of GDM.

In the last decade, there have been significant efforts to improve the outcome of pregnancy in women with GDM. Among these are various nutritional intervention strategies to reduce the risk of GDM. While considerable progress has been made, several challenges remain, including; non-compliance with dietary advice, reluctance to ingest tablets or use insulin injections, difficulty adhering to the treatment, and ongoing concerns over the long-term safety of oral agents on the mother and offspring.

Nutritional supplements are safe and generally well-tolerated and provide an alternative option for the treatment and prevention of GDM. However, studies examining the role of probiotics, omega 3 fatty acids, Myo-Inositol, vitamin D, selenium, zinc, and magnesium for the prevention of GDM are limited by the small sample size and the lack of reproducibility. The strength of the evidence for some commonly studied supplements is discussed in this narrative review focusing more on systematic reviews and meta-analysis. Besides, we will discuss the future directions for the prevention and treatment of GDM. A summary of the most recent systematic reviews and meta-analyses is included in Table 1.

**Table 1:** Summary of systematic reviews and meta-analyses of RCTs on the effects of nutritional supplementation during pregnancy.

**References**	**Study type**	**Intervention**	**Inclusion criteria**	**Significant results**
**Probiotics**
Chen et al. (8)	Meta-Analysis (7 RCTs)	Probiotic supplementation	Pregnant women with GDM	Reduction in FBG and inflammatory markers
Zhang et al. (9)	Meta-analysis (11 RCT)	Probiotic supplementation	Pregnant women with GDM	Reduction in newborn's hyperbilirubinemia, maternal HDL- and inflammatory and oxidative stress biomarkers
Taylor et al. (10)	Meta-analysis) (4 RCT)	Probiotic supplementation	Pregnant women with GDM	Reduction in (HOMA-IR)
**Omega 3**
Amirani et al. (11)	Systematic review and meta-analysis (14 RCTs)	Omega-3 supplementation	GDM, overweight or obese women.	Improvement in HDL levels.
Middleton et al. (12)	Cochrane Review(70 RCTs)	Omega-3 supplementation	Pregnant women	Reduction in preterm delivery (<37 weeks); early preterm delivery (<34 weeks), & risk of low birth weight
**Vitamin D**
Ojo et al. (13)	Systematic Review and Meta-Analysis of 5 RCTs	Vitamin D supplementation	Pregnant women with GDM	Lower FBG, HbA1c, and serum Insulin concentration
Yin et al. (14)	Meta-Analysis (16 RCTs)	Vitamin D Supplementation	Pregnant women with GDM	Reduced FBG, reduced fasting Insulin, improved HOMA-IR, and HOMA-β, and (QUICKI)
Jin et al. (15)	Systematic Review and Meta-Analysis (13 RCTs)	Vitamin D supplementation	Pregnant women with GDM	Reduced FBG, and reduced HOMA-IR
**Myoinositol**
Crawford et al. (16)	Cochrane systematic review (4 RCTs)	Myo-Inositol supplementation	Pregnant women with GDM	Reduction in the incidence of GDM
Zhang et al. (17)	Systematic Review and Meta-Analysis (5 RCTs)	Myo-Inositol supplementation	Pregnant women with GDM	Reduction in the incidence of GDM and preterm delivery

## Probiotic Supplementation

Microorganisms are found in human tissues and bodily fluids, including the skin, mouth, saliva, nose, vagina, seminal fluid, and mammary glands. Most of them dwell in the gut due to nutrient accessibility (18). The human gut microbiota is believed to exceed the total cells in the human body by a factor of ten, including roughly 100 trillion microorganisms representing 5,000 different species (19). The gut microbiota plays a fundamental role in many physiological processes such as digestion, immunity, neurological signaling, endocrine function, drug and toxins metabolism, and producing secondary compounds that influence the host physiology (20).

The bacterial phyla *Bacteroidetes* and *Firmicutes* phyla dominate the gut microbiota in healthy adults (21). The gut microbiota also plays a central role in producing short-chain fatty acids (SCFAs), such as acetate, butyrate, and propionate, via fermentation of non-digestible dietary fibers (22). SCFAs can activate G-coupled receptors and stimulate the secretion of Glucagon-like peptide 1 (GLP-1) and Peptide YY from the L-cells. It increases leptin secretion from adipocytes increases both insulin secretion and insulin sensitivity and satiety (23). There is a growing body of evidence regarding the association between gut microbiota and metabolic disorders, including obesity, T2DM, and GDM. Dysregulation of the gut microbiota, known as dysbiosis, reduces the release of SCFA and other antimicrobial molecules (20). These changes can lead to dysregulation in insulin secretion, insulin sensitivity, and appetite regulation. Most critical is the increase in gut wall permeability, allowing endotoxins' systemic entrance, resulting in low-grade inflammation. Women with GDM have altered gut microbiota compared to normal glucose tolerance (24).

Probiotics are defined by the World Health Organization (WHO) as “live microorganisms which when administered in adequate amounts confer a health benefit on the host” (21). They are reported to improve microbial balance in the gastrointestinal tract, increase the colonic microbial diversity, improve intestinal barrier function (25, 26), attenuate inflammation and regulate insulin production (27). Additionally, probiotic supplementation during pregnancy modulates gut microbiota composition and improves glucose and lipid metabolism (28). Experimental and clinical evidence support that this effect could be beneficial in preventing GDM (29). Furthermore, combination probiotics (Lactobacillus and Bifidobacterium strains) during pregnancy reduces the risk of inflammatory events and preeclampsia and improves maternal glucose metabolism (30, 31).

A meta-analysis by Chen et al. (8), which included seven studies, showed that probiotic supplementation reduced fasting glucose (FBG) (WMD:−3.19 mg/dl, 95% CI:−5.55 to−0.82, *P* = 0.008) in pregnant women with GDM. This association was more significant in patients with a baseline FBG ≥ 92 mg/dl, a duration of probiotic treatment ≤ 6 weeks and a dose <6 × 10^9^ colony-forming unit (CFU). In addition, the supplementation was effective in reducing some of the biomarkers of inflammation and oxidative stress like high-sensitivity CRP and malonaldehyde. Similarly, a meta-analysis of 11 randomized controlled trials assessed the effects of probiotic supplementation for 4 to 8 weeks showed that probiotic supplementation in women with GDM reduced the incidence of a newborn's hyperbilirubinemia and improved maternal HDL-cholesterol and markers of inflammation and oxidative stress (9).

Conversely, other studies did not support these findings and showed that probiotics did not prevent GDM in overweight and obese pregnant women (32, 33). Another meta-analysis showed that 6–8 weeks of probiotic supplementation significantly reduces the homeostasis model of assessment-estimated insulin resistance (HOMA-IR) but did not affect the FBG or LDL-cholesterol (10).

One explanation of the above disparity could be that probiotics differ in their ability to resist gastric acid and bile acids colonizing the intestinal tract (34). Therefore, not all probiotics exert similar clinical benefits; whether used as a single or combination of species (35), this could explain the un-unifying evidence about their impact on GDM prevention and maternal glucose metabolism. Besides, the individual response to the treatment may play a role. Hence, personalized and precise supplementations should be tested, considering interaction with diet and the host gut microbiota composition during pregnancy.

In summary, gut microbiota dysbiosis associated with inflammation, adiposity, and glucose intolerance, was observed in women with GDM, which resembles the gut microbiota profile of adults with other metabolic disorders such as T2DM. Microbiota-targeted interventions such as probiotics/prebiotic or combination symbiotic supplementation have already shown promising results and could enhance health outcomes in women with GDM.

A combination of lifestyle changes like exercise and diet, microbiota targeted, and novel approaches should be considered and tested, considering the side effects of such intervention. Thus, long-term studies using multi-strain probiotics/postbiotic or engineered microbes are warranted involving large GDM cohorts from different ethnicities.

## Fish Oils and Fatty Acids Supplementation

Fish are rich in long-chain polyunsaturated fatty acids. The relationship between fish intake and metabolic disorders is controversial (36). While white and oily fish was associated with a reduced risk of T2DM, fried and shellfish were associated with a higher risk of T2DM (37). Besides, the relationship between T2DM and fish intake varies by gender and geographical location (38). In pregnancy, there is more focus on the mercury contents of fish, and there are no uniform recommendations on fish intake (39). However, fish are not the only source of long-chain polyunsaturated fatty acids. Omega-3 polyunsaturated fatty acids (O-3-PUFA) [Eicosapentaenoic acid (EPA) and Docosahexaenoic acid (D.H.)] are also derived from plants, such as leafy greens, seeds, and nuts (40). O-3-PUFA modulates inflammatory pathways and exert anti-inflammatory and anti-coagulant effects (40). Furthermore, O-3-PUFA has several potential benefits, including antilipidemic, anti-hypertensive effects; modulation of gut microbiota; regulation of satiety; and enhancement of insulin sensitivity (40–42). In the growing fetus, 0-3-PUFA are critical for the structural and the functional development of many organs, particularly the brain and the eyes (43).

The antilipidemic effects of O-3-PUFA are of particular interest in pregnancy with or without GDM. Pregnancy impairs lipid metabolism and alters the serum concentration of free fatty acids (FFA), triglycerides (T.G.), total cholesterol (T.C.), High-density lipoprotein (HDL), and Low-density lipoprotein (LDL) (44). Indeed, there is a steady rise in T.G. and lipoproteins from the eighth week of gestation (44). GDM further aggravates lipids metabolism. Compared to women with normal glucose tolerance, women with GDM had higher levels of T.G., dense LDL particles, T.C., similar levels of LDL, but lower HDL levels (45). There is a shred of growing evidence that, in GDM, maternal T.G. levels positively correlate with neonatal weight and the risk for LGA and macrosomia (46–48). Furthermore, higher T.G. levels were found to worsen GDM- associated endothelial dysfunction in the umbilical vein (49).

Outside pregnancy, treatment with Omega-3 (EPA and D.H.) at a dose of 4 g/day were shown to be “an effective and safe option for reducing triglycerides as monotherapy” (50). Lower doses were not shown to be effective in reducing T.G. levels. Recently, a purified form of EPA, Icosapent ethyl (at a dose of 4 g/day), was shown to reduce the composite of cardiovascular death, non-fatal myocardial infarction, or non-fatal stroke in patients with established cardiovascular disease and elevated T.G. levels (51).

In pregnancy, most studies did not show a benefit of Omega-3 supplementations, likely due to (i) low dose of Omega-3 used in the trials, (ii) short duration of exposure to the treatment, and (iii) the small sample size. A meta-analysis of 14 RCT showed favorable effects of Omega-3 treatment on HDL but not on T.G., HOMA-IR, and fasting glucose (11). Of the 14 studies, only one used a dose of 4 grams; most studies lasted for <20 weeks and enrolled <100 subjects (11). Some studies have shown limited benefits from Omega-3 in women with GDM. Supplementation of Omega-3 fatty acid (at a dose of 1 g per day) in 52 women with GDM had some beneficial effects on insulin resistance but did not impact plasma glucose (HOMA-B), QUICKI, and lipids profile (52). Co-supplementation of omega-3 fatty acids (at a dose of 2 g per day) and vitamin D for 6 weeks in 140 women with GDM improves fasting glucose, insulin levels, serum triglycerides, and VLDL cholesterol (49). An RCT of 40 women with GDM showed that supplementation of omega-3 fatty acids (at a dose of 2 g per day) for 6 weeks significantly improved gene expression of peroxisome proliferator-activated receptor-gamma (PPAR-γ), interleukin-1 (IL-1), and tumor necrosis factor-alpha (TNF-α) (53). Co-supplementation of omega-3 fatty acids (at a dose of 1 g per day) and vitamin E for 6 weeks in 60 women with GDM did not improve insulin resistance or lipids but improved plasma total antioxidant capacity plasma malondialdehyde, and nitric oxide (54). A study of the long-term influence of GDM and the effect of omega-3 polyunsaturated fatty acids on the pancreas of the offspring found that supplementation lowered the pancreatic oxidative stress and inflammation, as evident by pancreatic fatty infiltration (55).

A recent Cochrane review has shown other potential benefits of Omega-3 supplementation in pregnancy (12). The Cochrane review included 70 RCTs involving 19,927 pregnant women showed a high risk of attrition bias in most studies. The use of Omega-3 supplementation showed a reduction in preterm delivery (<37 week's gestation), early preterm delivery (<34 week's gestation), and the risk of low birth weight.

In summary, the current evidence is insufficient to recommend for or against the routine supplementing of pregnant women with Omega-3. However, there are multiple potential benefits in pregnant women with or without GDM that should be examined in RCT. We recommend that future trials start at an early gestational age, be sufficiently powered, and use 4 g of Omega-3.

## Vitamin D Supplementation

Vitamin D (Vit D) deficiency is not uncommon amongst pregnant women and has been linked with some pregnancy complications such as GDM, preeclampsia, preterm birth, and small for gestational age (56). The true prevalence of Vit D deficiency during pregnancy is ill-defined as different cut-off levels of 25-hydroxyvitamin D level (25 OH D) was used in studies. A study from the Middle East and North Africa (MENA) region found that the prevalence of Vit D deficiency amongst pregnant women [defined as 25 (OH) D <50 nmol/l] ranged between was 54 and 90% (57). Saraf et al. (58) meta-analysis found that the global prevalence of Vit D ranged between 46 and 87% for cut off levels of 25(OH) D <50 nmol/; and between 9 and 79% for cut off levels <25 nmol/l (59).

Many studies have shown an association between Vit D deficiency and increased risk of GDM (59–66), while others did not show similar results (67). Furthermore, high serum 25(OH) D levels (>81 nmol/l) were found to be protective against GDM when compared to moderate-high Vit D levels (63–81 nmol/L, RR: 0.47; 95% CI: 0.23, 0.96) (68).

Vit D supplementation can improve glucose metabolism in women with GDM by lowering FBG, HbA1c, and serum insulin concentration (13). A meta-analysis showed that Vit D supplementation significantly reduced FBG (SMD =-1.87, 95% CI-3.39-0.35) and the incidence of GDM (OR = 0.42, 95% CI 0.30–0.60) in pregnant women. It also showed that Vit D supplementation significantly reduced FBG insulin levels, improved the HOMA-IR and HOMA-β, and increased the quantitative insulin sensitivity check index (QUICKI) in women with GDM (14). However, a multicentre European study (The DALI vitamin D randomized controlled trial for GDM prevention) showed no significant benefit from Vit D intervention besides improving vitamin D status (69). In this trial, pregnant women were randomized into eight groups; healthy eating (HE), physical activity (PA), HE & PA, HE& PA + Vit D3 1600 IU/day, HE& PA + placebo, Vit D3 1600 IU/day, and placebo. While 98% of the subjects enrolled on Vit D arms achieved a serum 25(OH)D ≥ 50 nmol/l, there was no reduction in the risk of GDM despite a small but significantly lower FPG (-0.14 mmol/l; CI95 −0.28, −0.00) compared to placebo (69).

Supplementation of Vit D in combination with other supplements have also been studied during pregnancy. Aside from improving insulin resistance markers in women with GDM, Vit D and evening primrose oil significantly reduced serum triglycerides, VLDL, total cholesterol, and LDL concentrations (70). Vitamin D-magnesium-zinc-calcium co-supplementation to women with GDM for 6 weeks was shown to reduce inflammation and oxidative stress biomarkers (CRP and plasma malondialdehyde concentrations) and to increase total antioxidant capacity levels compared to placebo. In addition, this study also found a decreasing trend in birth weight and the rate of macrosomia (3.3 vs. 16.7%, *P* = 0.08) (71).

A recent meta-analysis illustrated that vit D supplementation significantly reduced FBG and regulated HOMA-IR and that vit D supplementation was superior to omega-3 (-3.64 mg/dL, 95% CI:-5.77 to-1.51), zinc (-5.71 mg/dL, 95% CI: -10.19 to -1.23), probiotics (-6.76 mg/dL, 95% CI:-10.02 to -3.50), and placebo (-12.13 mg/dL, 95% CI:-14.55 to -9.70) for improving FBG (15).

Personalized variations in response to different treatments among different study populations are expected due to genetics and lifestyle factors, a concept that has been increasingly supported in this era of precision medicine (72). Given the evidence presented above, Vit D supplementation may be beneficial in preventing and treating GDM. Hence, assessing vitamin D status in early pregnancy may be clinically helpful for risk assessment and developing effective and personalized interventions for the prevention and treatment of GDM.

## MYO-Inositol Supplementation

Myo-Inositol is the most abundant isomer of Inositol, a naturally occurring polyol sugar commonly found in cereals, beans, nuts, meat, legumes, and fresh citrus fruits. Myo-Inositol and Chiro-Inositol are inositol precursors, known as inositol phosphoglycans (IPGs) (73).

Myo-Inositol is a component of the membranes of all living cells and can be produced by the human body from D-glucose (74). It also plays a role in synthesizing lipids and occurs in its free form as a component of phospholipids or as phytic acid. It is one of the intracellular mediators of the insulin-signaling pathway and correlates with insulin sensitivity in T2DM (74). It is an insulin-sensitizing mediator, which is reported to reduce plasma glucose levels improve insulin sensitivity and ovulatory function in young women with PCOS (75). Myo-Inositol is needed for both production and activation of PI3 Kinase, essential for normal cell glucose metabolism (76). In patients with PCOS, Myo-Inositol restored glucose uptake, similar to Metformin, through an AMPK activation leading to an increase in GLUT-4 levels and glucose uptake by human endometrial cells (77). It has also been reported that conditions associated with insulin resistance are characterized by a high level of urinary inositol metabolites (78).

A prospective RCT included pregnant women with a parent who had T2DM but not those who were obese or had a history of PCOS, GDM, or pre-gestational diabetes. From the end of the first trimester, patients were randomly assigned to either 2 g Myo-Inositol and 200 mcg folic acid twice a day or 200 mcg folic acid twice a day. Myo-Inositol supplementation reduced the incidence of GDM (6 vs. 15.3 %, *P* = 0.04) and the incidence of macrosomia (79). When given at the end of the first trimester, Myo-Inositol also reduced the incidence of GDM (14 vs. 33.6%) in obese women (80). A Cochrane systematic review of four randomized controlled trials of 567 Italian women; most studies had small sample sizes, and two were open-label. The Cochrane review showed that Myo-Inositol was associated with a reduction in the incidence of GDM [risk ratio (RR) 0.43, 95% confidence interval (CI) 0.29 to 0.64]. However, there was no consensus on the neonatal outcomes (16). A more recent systematic review and meta-analysis of 5 RCTs showed that Myo-Inositol was associated with a significant reduction in the incidence of GDM and preterm delivery. However, Myo-Inositol supplementation had no impact on 2-h glucose of oral glucose tolerance test, gestational age at birth, birth weight, or macrosomia (17).

In conclusion, Myo-Inositol's potential benefit in improving insulin sensitivity suggests that it may help prevent GDM in obese, overweight women, women with PCOS or a family history of T2DM. However, large RCTs in different ethnic groups and more comprehensive risk factor profiles are required before Myo-Inositol could be recommended to prevent GDM.

## Other Supplements

Magnesium supplementation improves glucose metabolism in people with diabetes and improves insulin sensitivity parameters in those at high risk of diabetes (81). In addition, Magnesium supplementation for 6 weeks in women with GDM has been shown to have beneficial effects on the expression of inflammatory markers and genes related to insulin and lipid metabolism (82, 83). Ultimately, this might decrease metabolic complications in women with GDM. A recent meta-analysis demonstrated that omega-3, magnesium, Vit D, zinc, and probiotics improved FBG, serum insulin, and HOMA-IR compared to placebo. Magnesium supplementation was superior to other supplements in decreasing serum insulin (15).

Zinc and selenium are trace elements required for the activity of glutathione peroxidase and other antioxidant functions (84). The level of the two supplements is reported to be low during pregnancy (85). A meta-analysis showed that serum selenium concentration is significantly lower in women with GDM compared to normoglycemic women (86). On the other hand, there was no difference in the zinc status between women with and without GDM (87). Overall, there is insufficient evidence that zinc supplementation during pregnancy improves maternal or neonatal outcomes (88).

There is no evidence to support an association between iron supplementation and the risk of GDM. On the contrary, pregnant women with iron deficiency anemia are less likely to develop GDM, as suggested by a meta-analysis of six studies including over 15,000 women (OR 0.61; 95% CI 0.47–0.80; PA = 0.0003) (89). A meta-analysis by Kataria et al. (90) including over 30 studies investigating the association of iron biomarkers and dietary iron exposure with GDM, demonstrated high iron biomarkers associated with GDM. Due to the high heterogeneity of the analyses, these findings should be interpreted with caution. Overall, there is no conclusive evidence linking routine iron supplementation in non-anemic women to an increased risk of GDM.

Finally, because the studies were conducted in different populations with different ethnicities and socioeconomic backgrounds, it is vital to approach all evidence of these supplements' association with GDM with caution. Well-designed prospective studies are required to understand the dynamic relationship between these minerals and GDM risk.

## The Current Position and Summary of the Evidence So Far

**Figure 1:**
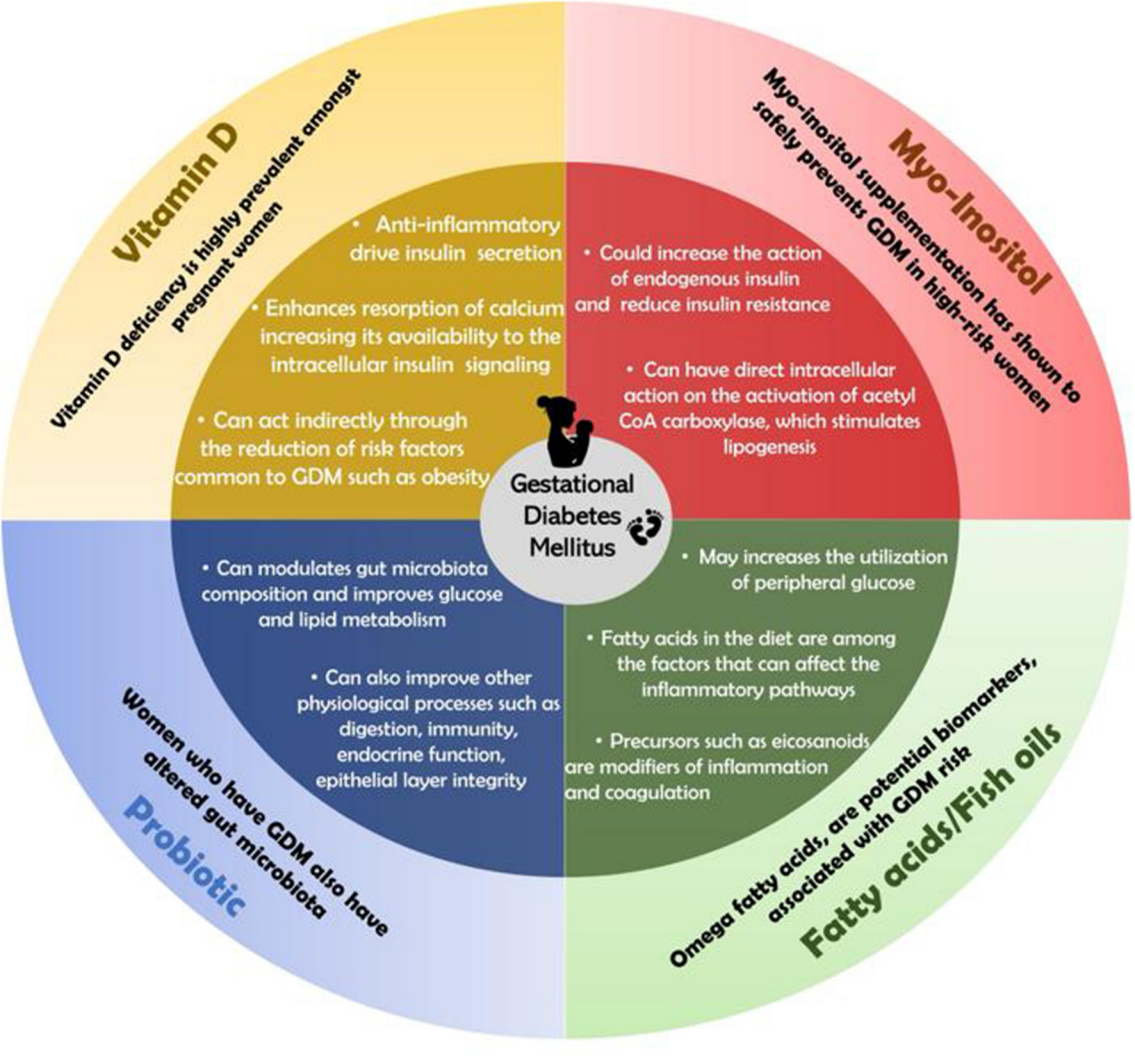
The effect of nutritional supplementation on glucose metabolism during pregnancy.

The use of vitamin D, Myo-Inositol, Probiotics and Omega 3 fatty acids in pregnancy is associated with a reduction in systemic inflammation and lipolysis, improvement in Insulin resistance and Insulin signaling through direct or indirect mechanisms. Thus, the use of these supplements appears to improve glucose haemostasis in women with or without GDM, which is summarized in Figure 1. Indeed, a recent meta-analysis published in 2020 found that omega-3, magnesium, Vit D, zinc, and probiotics were more effective than placebo in improving FPG, serum insulin, and HOMA-IR. FPG was significantly reduced, and HOMA-IR was regulated by vitamin D supplementation. Magnesium supplementation was more effective than other nutrient supplements in lowering serum insulin levels (15).

A recent randomized controlled, double-blind trial investigated the effect of Myo-Inositol, probiotics, and multiple micronutrients on gestational normoglycemia and preterm birth during the preconception and antenatal period (91). There were no differences between the intervention and control groups in any glucose measurements during the OGTT, nor in the incidence of GDM, birth weight, or gestational age at birth. This trial found a reduction in the incidence of major postpartum hemorrhage independent of cesarean section rates, parity, or birth size, as well as a potential benefit of Myo-Inositol-containing supplements in reducing preterm birth (91). Multi-ethnic women from three continents participated in this study. However, certain ethnicities were underrepresented, with less than half of the participants being overweight or obese. It did not analyze data separately for each ethnicity and relied on sachet counts to assess adherence to Myo-Inositol supplementation.

The summary of the evidence for the different interventions comes from a very recent Cochrane database of systematic reviews (92). It demonstrated that there was unknown benefit or harm of the following interventions on the risk of GDM: dietary advice vs. standard care, a low glycaemic index diet vs. a moderate-high glycaemic index diet, probiotic with dietary intervention vs. placebo with dietary intervention, Vit D, and calcium supplementation vs. placebo, and exercise interventions vs. standard antenatal care. On the other hand, there was a possible benefit of combined diet and exercise interventions during pregnancy vs. routine care (RR 0.85, 95% CI 0.71 to 1.01). There was also possible evidence of benefit for Myo-Inositol and Vit D supplementation during pregnancy vs. control in reducing the risk of GDM (RR 0.43, 95% CI 0.29 to 0.64 and RR 0.51, 95% CI 0.27 to 0.97, respectively) (91). However, there was clear evidence of no effect for omega-3 fatty acid supplementation on GDM risk.

## Future Directions

The high prevalence of GDM and the associated maternal and perinatal morbidity significantly strain current and future healthcare resource utilization. More importantly, GDM remains an important surrogate marker for developing T2DM in the future. As a result, pregnancy provides a unique and critical window of opportunity for interventions to reduce the long-term burden of diabetes. Antenatal supplementation with Myo-Inositol, vitamin D, and probiotics to prevent GDM is a relatively new and novel intervention. These readily available supplements as single agents or in combination are approved food supplements. Although the limited emerging evidence indicates that their use may be beneficial in reducing the incidence of GDM, further studies from different ethnic contexts and with differing risk factors are needed to assess its effects on maternal and neonatal outcomes. Given their availability as dietary supplements and their relatively low cost compared to traditional interventions for preventing GDM, exploring its potential role in reducing GDM is a much needed and timely study for high-risk populations. In the same context, a double-blind, randomized controlled trial is currently underway at Sidra Medicine, Doha, Qatar, to examine the effect of antenatal dietary Myo-Inositol supplementation on the incidence of gestational diabetes mellitus and fetal outcome (93). We believe the results from this study will contribute to the generalizable knowledge and direction of future research on GDM.

Before drawing meaningful conclusions about the effects of dietary supplements on maternal glycemia, we need to look at different subgroup populations, different nutritional supplement dose regimens, and the appropriate time for starting such supplements.

The assessment of longitudinal changes in Myo-Inositol levels and other nutritional supplements may help define potential pathways of effect and should be considered in the future when designing more definitive trials.

## Author Contributions

All authors have contributed to the literature review, writing the manuscript, and agreed to the published version.

## Conflict of Interest

MB was employed by Hamad Medical Corporation. The remaining authors declare that the research was conducted in the absence of any commercial or financial relationships that could be construed as a potential conflict of interest.

## Publisher's Note

All claims expressed in this article are solely those of the authors and do not necessarily represent those of their affiliated organizations, or those of the publisher, the editors and the reviewers. Any product that may be evaluated in this article, or claim that may be made by its manufacturer, is not guaranteed or endorsed by the publisher.
